# Goffin's Cockatoos (*Cacatua goffiniana*) Can Solve a Novel Problem After Conflicting Past Experiences

**DOI:** 10.3389/fpsyg.2021.694719

**Published:** 2021-06-29

**Authors:** Katarzyna Bobrowicz, Mark O'Hara, Chelsea Carminito, Alice M. I. Auersperg, Mathias Osvath

**Affiliations:** ^1^Department of Psychology, Lund University, Lund, Sweden; ^2^Department of Philosophy and Cognitive Science, Lund University, Lund, Sweden; ^3^Messerli Research Institute, University of Veterinary Medicine Vienna, Vienna, Austria; ^4^Royal (Dick) School of Veterinary Studies, University of Edinburgh, Edinburgh, United Kingdom

**Keywords:** memory, tool use, innovation, Goffin's cockatoo, executive functions, flexibility

## Abstract

Novel problems often partially overlap with familiar ones. Some features match the qualities of previous situations stored in long-term memory and therefore trigger their retrieval. Using relevant, while inhibiting irrelevant, memories to solve novel problems is a hallmark of behavioral flexibility in humans and has recently been demonstrated in great apes. This capacity has been proposed to promote technical innovativeness and thus warrants investigations of such a mechanism in other innovative species. Here, we show that proficient tool—users among Goffin's cockatoos—an innovative tool—using species—could use a relevant previous experience to solve a novel, partially overlapping problem, even despite a conflicting, potentially misleading, experience. This suggests that selecting relevant experiences over irrelevant experiences guides problem solving at least in some Goffin's cockatoos. Our result supports the hypothesis that flexible memory functions may promote technical innovations.

## Introduction

In humans, long-term memories can guide problem solving, given sufficient overlap between a past and a new situation. Such overlaps can cue retrieval of experiences and prime behaviors that were productive under similar circumstances (Tulving and Pearlstone, 1966; Tulving, 1974). However, when several salient but irrelevant properties overlap and relevant properties are more subtle, the retrieved memory may be inappropriate for the immediate problem. For example, some problems might appear very similar, but in fact differ in mechanism or causal structure. In such cases, inappropriate memories should be suppressed in favor of memories of another situation, related to the causal structure and not to the visual similarity (Anderson and Spellman, 1996; Anderson et al., 2016). Such inhibition of irrelevant memories in favor of relevant ones is considered a hallmark of human memory flexibility (Anderson, 2005), and, arguably, supports behavioral flexibility, the ability to switch from a previously rewarding behavior when changes in the environment occur (Lea et al., 2020). Behavioral flexibility, in turn, supports technical innovativeness, an ability to innovate new tools and their applications, which seems to require a combination of previous experiences to solve a new problem (Call, 2013; Auersperg, 2015) and flexible adaptation to demands of the problem (Laumer et al., 2017).

Flexibility in physical problem solving draws on (1) matching perceptual cues in the present task with similar cues, experienced in the past, (2) combining previous experiences to guide responses in the present, and (3) adjusting one's own responses based on the perceptual feedback from the present task. As problem solving typically depends on perceptually-based relations (Povinelli and Henley, 2020), pitting the actual relevance against the apparent relevance of these relations provides a measure of flexibility in problem solving across species.

A recent study on an orangutan and chimpanzees suggests that great apes flexibly use relevant experiences to solve a novel problem, even if interrupted by irrelevant experiences (Bobrowicz et al., 2020). Across two experimental conditions, great apes generalized tool function despite irrelevant perceptually-based relations that were either distracting (no-conflict condition) or misleading (conflict condition).

In one (no-conflict) condition, the subject encoded a relation between a tool, parts of a puzzle box and an action, and thereafter applied this relation to another, perceptually dissimilar test box. That is, the subject needed to generalize tool function to the test box despite the potentially distracting, irrelevant perceptual details of the initial box. In turn, in another (conflict) condition, after encoding the relevant tool function on the initial box, the subject would encode an additional tool function on a puzzle box that looked misleadingly similar to the test box. Thereafter, the test box would cue the retrieval of two competing tool functions, embedded in two potentially relevant memories. To solve the test task, the tool function acquired on a similar-looking box must be inhibited in favor of the tool function acquired on a different-looking box. Therefore, in the conflict condition, the subject needed to generalize the relevant, functional aspects of the initial box despite previously relevant but now irrelevant, perceptual details of the misleading box.

As generalizations always happen in response to a present problem, they must draw on flexible memory retrieval. For instance, in the test task presented to the great apes (Bobrowicz et al., 2020), they needed to match the perceptual cues in the task with perceptual cues encoded in the past task(s) and retrieve the relation(s) that back then led to a successful solution. Such generalizations result from re-combinations of memory traces whose retrieval is triggered by currently available cues. The relevance of information bits is guided by perceptual and functional similarity and is estimated across a pool of experiences available in an animal's memory. As it was found that competition between cued, potentially relevant memories involves fronto-hippocampal inhibitory control in rats (Bekinschtein et al., 2018), we would expect that flexible problem solving that involves inhibiting misleading experiences draws on explicit memories. One must note that this does not necessarily require any second-order representations, or any overt or aware understanding of the process (e.g., representing the memories as memories) (Anderson and Spellman, 1996; Anderson, 2005; Anderson et al., 2016; Bekinschtein et al., 2018).

As great apes—our closest living relatives—are among the most innovative animals (Manrique et al., 2013; Call, 2015), it is likely that their technical innovativeness draws on the executive functions that underpin flexible memory retrieval. Whether this type of memory flexibility is linked to innovativeness across animal taxa, or is specific to hominoids, requires the study of phylogenetically distant, but similarly innovative species, for instance, Goffin's cockatoos.

Goffin's cockatoos (Cacatua goffiniana) have a highly developed innovative capacity (Auersperg et al., 2012; Rössler et al., 2020) and have repeatedly shown considerable optimization abilities and inhibition (Auersperg et al., 2008, 2013b). They also solve novel problems by recombining familiar ones and are sensitive to both functionally relevant and irrelevant aspects of such problems (Auersperg et al., 2013a). Notably, they can innovate several modes of tool use [10, 16], which is important from a comparative perspective as ape memory flexibility was tested in a tool-using context (Bobrowicz et al., 2020). Therefore, Goffins are ideal non-hominoid candidates to investigate memory flexibility.

The experimental paradigm replicated the one used on great apes and, likewise, consisted of a sequence of three tool—use tasks. The third—the test task—required tool function encoded on a perceptually dissimilar task but looked similar to another task that required another, now irrelevant, tool function. Therefore, like in the great apes (Bobrowicz et al., 2020), the test task would provoke competition between these two tasks, in which the relevant but perceptually dissimilar memory would compete with the perceptually similar, but in fact functionally irrelevant memory. In practice, this competition would boil down to two tool-motor action pairings, where the relevant one is encoded on a different-looking task and the irrelevant one is encoded on a similar looking task. To arrive at a correct solution, the cockatoos would need to inhibit the seemingly relevant tool-motor action pairing acquired on a similar-looking task and instead apply the truly relevant tool-motor action pairing, even though it was acquired on a different-looking task.

A 24-h delay separated the first two tasks from the test task to ensure testing the resolution of competition between long-term memories. Based on high levels of behavioral flexibility and technical innovativeness in Goffin's cockatoos, we expected that Goffin's cockatoos may have access to flexible memory retrieval. Therefore, we expected that at least some Goffins would solve the test task in both experimental conditions, performing on par with great apes. We also expected that the successful Goffins' interactions with the test task would follow a similar pattern to the interactions of successful great apes, suggesting that similar cognitive functions may underpin such interactions.

## Materials and Methods

### Subjects

Twelve adult Goffin's cockatoos (4 females, *Cacatua goffiniana*) participated. They were housed in a social group of sixteen birds in a large aviary at the Goffin Lab in Goldegg, Lower Austria, associated with the Messerli Research Institute at the University of Veterinary Medicine Vienna. Three juvenile birds in the group were too young to participate in the study and one adult bird was excluded from the study due to a sustained neophobic reaction to the apparatus. The subjects had previous experimental experience, including problems that required manufacturing and re-shaping tools to reach a distant, otherwise out-of-reach, reward (Auersperg et al., 2008, 2012, 2014, 2016; Laumer et al., 2017). These previous setups did not require tools and motor actions involved in the current study. This study was conducted prior to a recently published study with the same subjects, involving partially similar apparatuses (Rössler et al., 2020).

All subjects used in this experiment originate from accredited European breeders, have full CITES certificates and are officially registered (following the Austrian Animal Protection Act §25 - TschG. BGBl. 118) at the district's administrative animal welfare bureau (Bezirkshauptmannschaft St. Polten Schmiedgasse 4-6, A-3100; St. Poten, Austria). The housing conditions, described here and in detail in the Supplementary Information, are in accordance with the species-specific guidelines provided by the Austrian Federal Act on the Protection of Animals (Animal Protection Act -§24 Abs. 1 Z 1 and 2; §25 Abs. 3 – TSchG, BGBl. I Nr. 118/2004 Art. 2). Furthermore, as our experimental procedures were purely appetitive, strictly non-invasive and based exclusively on behavioral tests, they were classified as non-animal experiments in accordance with the Austrian Animal Experiments Act (§2. Federal Law Gazette No. 501/1989), according to which ethical approval is not required for this study. The birds are not wing-clipped and therefore partake in experiments voluntarily: they are called into the experimental chamber by name. Various fresh and cooked food sources and fresh water is always available *ad libitum* in our aviary. Specific treats (cashew nuts) are reserved for experiments.

### Experimental Design

This study had a mixed design, with three factors varied across subjects: condition, order of conditions and task set (see Table 1). Overall, three conditions were introduced to provide subjects with either no relevant experiences (control), a relevant experience (no-conflict) or a relevant and an irrelevant experience (conflict) before attempting to solve a test. Each subject was randomly assigned to two different conditions, that is, control and no-conflict, control and conflict, or no-conflict and conflict (except for Dolittle, see Table 1 and Supplementary Information 1). Two sets of tasks were needed, so that the subject would not repeat the same tasks in both conditions. This means that each subject completed two task sets across the two experimental conditions. Therefore, task set was a within-subject factor, and condition and order of conditions were between-subject factors. The order of conditions was pseudorandomized across individuals.

**Table 1:** An overview of the experimental design.

**Group**	**Name**	**Set**	**Condition**	**Score in the test**
Hookset first	Dolittle	Hookset	Conflict	0
		Screwset	Conflict	1
	Fini	Hookset	Control	0
		Screwset	No-conflict	1
	Kiwi	Hookset	Conflict	0
		Screwset	Control	0
	Konrad	Hookset	Control	0
		Screwset	Conflict	Did not pass the FOT
	Mayday	Hookset	No-conflict	0
		Screwset	Conflict	Did not pass the FOT
	Muppet	Hookset	No-conflict	0
		Screwset	Control	0
Screwset first	Figaro	Hookset	No-conflict	1
		Screwset	Conflict	1
	Heidi	Hookset	Conflict	0
		Screwset	Control	0
	Moneypenny	Hookset	Control	Did not pass tool familiarization
		Screwset	Conflict	Did not pass the FOT
	Muki	Hookset	No-conflict	0
		Screwset	Control	0
	Pipin	Hookset	Conflict	Did not pass tool familiarization
		Screwset	No-conflict	1
	Zozo	Hookset	Control	0
		Screwset	No-conflict	0

*Each subject (except for Dolittle) was assigned to two out of three available conditions*.

### Apparatus

Initially, subjects received each task in an experimental setup that consisted only of a puzzle box and tools, a wooden base, and a flat surface on which the birds could walk freely throughout the experiment. However, to prevent haptic exploration of the puzzle boxes, a large cage was introduced (h75 cm × w75 cm × d43 cm; openings: h1.7 cm × w1.7 cm; h = height, w = width, d = depth; Supplementary Figure 1). The cage was divided into two compartments by inserting a mesh wall (h48.7 cm × w71 cm) that divided the interior of the cage into two compartments: one available to the subject, and another, in which the puzzle box was placed, available only to the experimenter. The mesh wall had large enough openings (h3 cm × w3 cm) that allowed inserting the tools to reach the puzzle box. Because sticking tools through these openings was motorically more difficult than using them directly on the puzzle box, we ensured that each bird was either participating in the same setup at all stages of the experiment or only had a baseline without the cage (for details see Supplementary Table 1).

**Figure 1:**
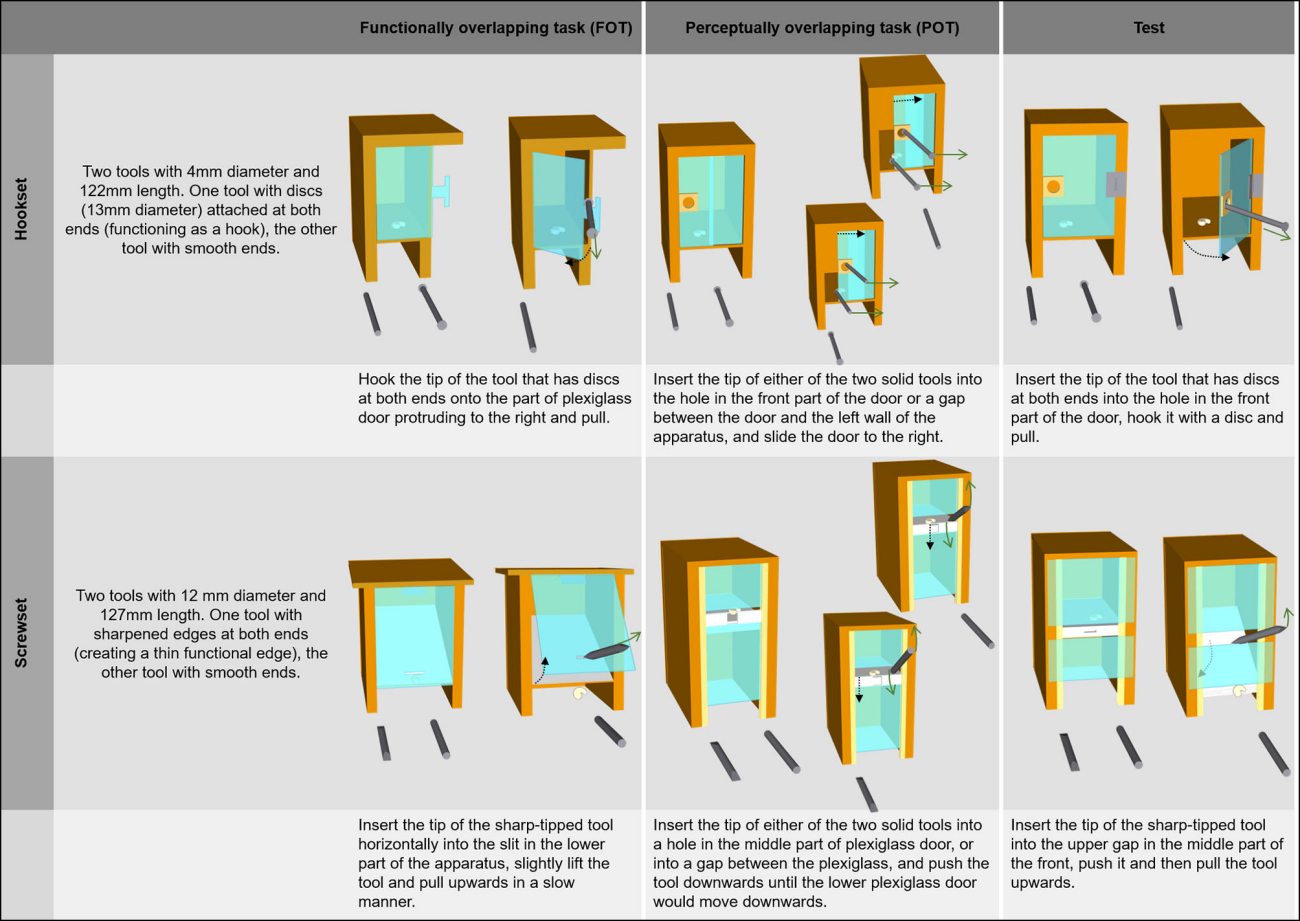
Schematic overview of tasks and required actions. Shown are initial setup for each task and end state after opening. Description of required actions are listed below the schematics. For the perceptually overlapping tasks solutions with either tool and different techniques are provided. Dotted arrows indicate movement direction of relevant apparatus parts, whereas solid arrows show required movement of tools.

**Figure 2:**
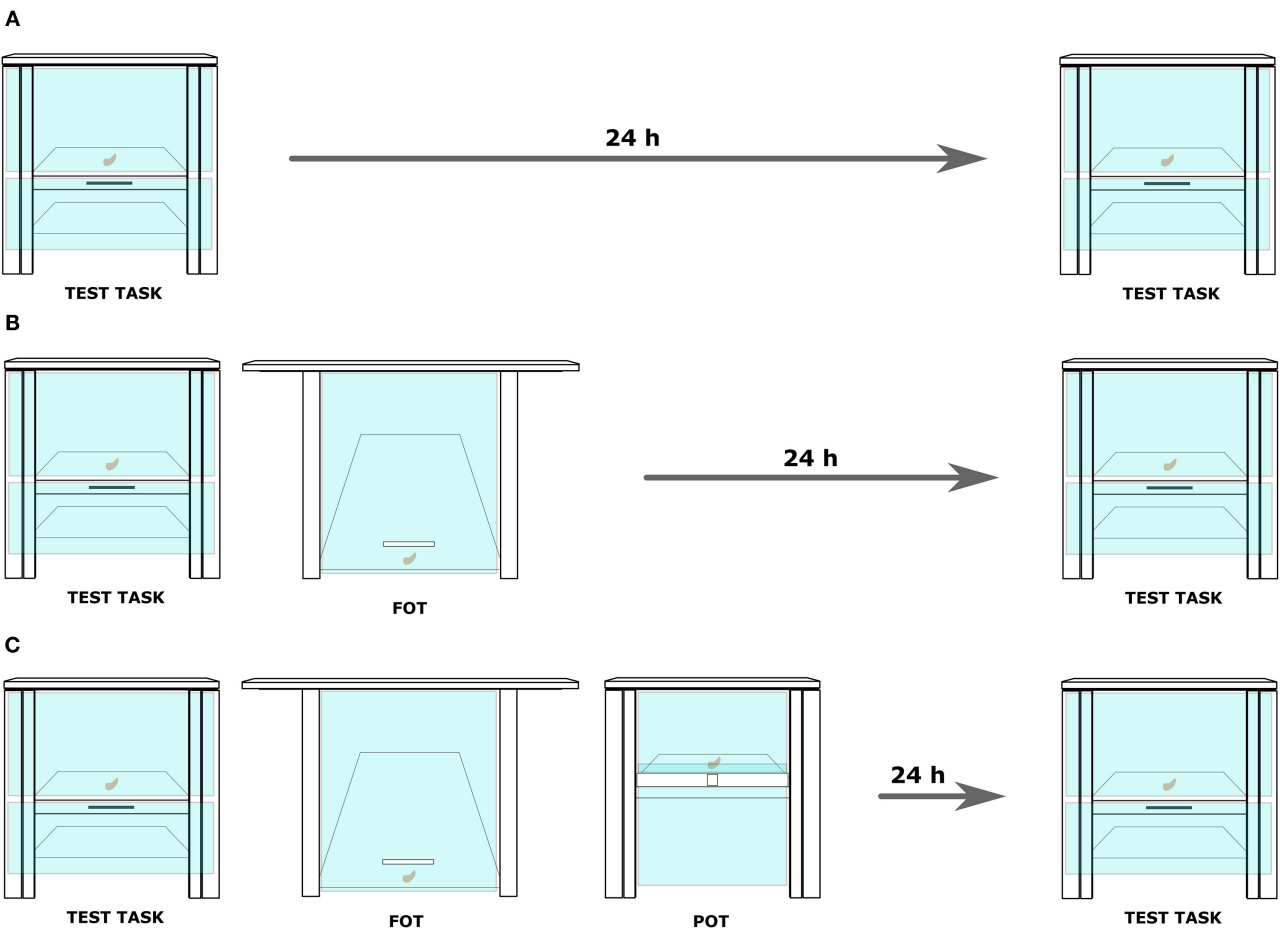
A display of three conditions introduced in the experimental setup. **(A)** Control: the subject was exposed twice to the test task. A 24-hour delay, but no trainings were conducted between the two exposures. **(B)** No-conflict: after the first exposure to the target problem, the subject received training on a functionally overlapping task (FOT). After it reached the learning criterion, a 24-h delay commenced before the second exposure to the test. **(C)** Conflict: in this condition, the training on the FOT was immediately followed by a training on a perceptually overlapping task (the POT). Again, after the learning criterion was reached, a 24-h delay commenced before the second exposure to the test.

Each task involved a puzzle box. Two sets of tasks, hookset and screwset, were used, and each set consisted of (1) a functionally overlapping task (FOT), (2) a perceptually overlapping task (POT), (3) a test task, and (4) three tools (Figure 1, Supplementary Figure 2 and Supplementary Information 2). All boxes were made from plywood and contained a visible food reward (a piece of a cashew nut) behind a transparent plexiglass surface (Figures 1, 2). For all dimensions of the boxes see Supplementary Figure 2.

Each set of the puzzle boxes was accompanied with three tools: a right tool, a wrong tool, and a useless tool. The right tool had two functional tips that allowed for insertion or hitching. The wrong tool lacked the functional tips but was otherwise identical to the right tool in both material and dimensions. The useless tool had the appropriate length but was not rigid enough to be functional. All tools used in a given trial were placed side by side in front of the puzzle box, in a random order across trials and birds.

All tools were made either of hard wood or baked modeling clay, and to minimize potential material preferences, the right tool and the wrong tool in a given trial were always made from the same material. The right tool in the hookset was made of a thin hard wood stick with small metal disks on both ends, to make sure that the tool was light enough to be held in the beak and strong enough to preclude tool destruction. The right tool in the screwset was initially made of hard wood but was later switched to modeling clay and plastic because of tool destruction.

To retrieve the reward from a given puzzle box, the subject had to choose a tool and apply it to the functionally relevant components of the box. Only components involved in opening the box and their immediate vicinity (+1 cm in each direction), were defined as relevant for the solution; other components were defined as irrelevant. The configuration always comprised fewer relevant than irrelevant components and varied between the boxes (Supplementary Figure 3). Once opened, the door either hinged outward toward the subject (hookset: the test and the FOT, screwset: the FOT) or slid upwards/to the side (in both POTs), or hinged downwards as a part of a trap mechanism (screwset: the test) (Figure 1).

The interaction that would lead to opening a given puzzle box always required three actions. The first action involved either (1) inserting the tip of the tool into a gap, or (2) hooking the tip behind a surface. The second action required stabilizing the tool in a fixed position, and the third action involved either pulling the tool (hookset: FOT and test) or lifting the held tip of the tool (screwset: FOT and test) or pushing it to the side/downwards (hookset and screwset: POT; Figure 1). Two puzzle boxes were defined as functionally overlapping if their opening required the same first and third action; two puzzle boxes were defined as perceptually overlapping if both their shape and dimensions (height, width, length) were identical.

To introduce an overlap between the FOT and the POT and promote competition between the FOT and the POT in the test, the right tool was available in both tasks. The FOT and the test tasks were solvable only with the right tool, and the POT was solvable with both the right and the wrong tool. Therefore, the right tool was always relevant for the solution and the wrong tool was only relevant for the solution of the POT, but neither for the FOT nor the test. This is a central aspect of the study's design. The same tool was functional (the right tool) in both FOT and POT, in order to give it a higher associative strength than the tool that was wrong in the test (conflict condition). Therefore, the functional tool might have become intrinsically rewarding, but as it was rewarding in two different arrangements of perceptual features and two different types of actions, the solution of the task in the test would require the animal to switch between these arrangements and actions. This, in effect, would put greater demands on the explicit memories.

### Procedure

The experimenter would typically remain on her own side of the cage to conveniently re-bait the puzzle box through an opening in its back wall, but if the subject was losing interest in the puzzle box, the experimenter would go to the subject's side, insert arms in the cage until the subject would start interacting with the puzzle box anew. Whenever the subject pushed the tool all the way through the mesh wall to the experimenter's side, the experimenter put it back to the subject's side to make it accessible without visual or motor indication to any tool. Throughout the trial, the experimenter would focus her gaze on the top or the back of the puzzle box to avoid guiding subjects' choice of tools and components of the puzzle box.

Before the experiments, the birds were familiarized with the tools and habituated to the cage (details in Supplementary Tables 1, 2). Only after the subject voluntarily grabbed the tools and had no neophobic reaction toward the cage did it proceed to the baseline. Note that some subjects received the baseline in the cage, and some outside of the cage (Supplementary Table 1). In the baseline, the subject was first offered a food reward, and then exposed to the baited test task and a complete tool set (Figure 2). During the baseline, we would record the first five trials, each of which would start when the bird picked up the tool and would be terminated by the experimenter when the bird would attempt to destroy the tool/the puzzle box, or would drop the tool, or would solve the test task (although none of the subjects solved the test at baseline in the current study). If the subject lost interest in the puzzle box, but remained in the vicinity, the experimenter would knock on the top of the puzzle box to attract the subject's attention. We did this to give the less persistent and/or attentive birds equal opportunities to those birds that were more persistent and/or attentive. Having such a way of attracting bird's attention would be even more important later in the trainings that would typically take even up to 10–15 min per day, and would be quite taxing for the less attentive, or more distractible, birds. If the subject used the right tool correctly and retrieved the reward in the baseline, it would be excluded from further testing on the respective set, but none of the birds in the current study did so. We stipulated that five trials would represent optimal exposure, that is, long enough to manipulate the tool correctly, but short enough to reduce frustration leading to a lack of motivation in subsequent trials. Thereafter, the test task was removed.

Note that three experimental conditions were introduced: control, no-conflict, and conflict to manipulate the availability of relevant and irrelevant experiences in the test. In the control condition, subjects would not receive any trainings (neither relevant nor irrelevant). In the no-conflict condition, subjects would receive only a relevant training on the FOT, and in the conflict condition, subjects would receive both a relevant training on the FOT and an irrelevant training on the POT. Right after the baseline, subjects in the control condition were returned to the social group and retested on the test task after 24 h. In both the no-conflict and conflict conditions, the baseline was followed by a training on the FOT (for details see Supplementary Information 1), during which the subject had access to the right and the useless tool. The training consisted of alternating demonstrations by the experimenter and attempts by the subject, using the right tool. The training was concluded once the individual retrieved the food item independently five consecutive times. Subjects in the no-conflict condition were returned to the social group and received exposure to the test task after 24 h. Subjects in the conflict condition received additional training on the POT task with the complete tool set, immediately after the training on the FOT and following the same procedure. The training on the POT task again consisted of alternating demonstrations by the experimenter and attempts by the subject, using both the right and the wrong tool. The experimenter interchangeably used both tools during the demonstrations in order to ascertain that the subject would learn that both tools are equally functional on the POT task. Twenty-four hours after reaching the learning criterion, individuals were exposed to the test task again.

The subjects had unlimited access to all tools available in a given trial (Supplementary Tables 2, 3). Only some subjects engaged in tool destruction, and as they did, spare copies of the tool were supplied, or the material of the tool was eventually changed (for details see Supplementary Information 1). Some subjects tried to stick toes through the mesh to reach the apparatus (Supplementary Figure 1); in such cases the experimenter moved the apparatus away from the subject. Only if the subject stopped this behavior would the apparatus be moved back. The testing session was discontinued if: (1) the subjects started destroying the apparatus, (2) the subjects avoided the vicinity of the setup and materials for 10 min, (3) the subjects attempted to exit the experimental cage while showing signs of neophobia. In such cases, the testing was continued on the next testing day. Each bird received only one test trial, in which the bird could attempt to solve the test task. The data were collected by KB and CC, and all trials were video-recorded.

During the test trial, subjects had unlimited time to interact with the task, but this trial was terminated if individuals (a) solved the task, (b) removed all the tools, (c) engaged in tool/apparatus destruction, (d) repeated unproductive motor actions on irrelevant components for more than 80% of the interaction time, or (e) refused to interact with the tools (on three separate occasions; for details see Supplementary Table 3).

### Coding

Trials were video-recorded. The interactions with the apparatus were coded frame-by-frame in ELAN 4.9.3. An interaction was defined as duration between the onset and offset of physical contact between a tool held by the subject and a component of the puzzle box. Two aspects of the interaction were determined: (1) the tool used, right or wrong, or useless; and (2) the component of the apparatus touched, relevant or irrelevant (Supplementary Table 4).

Two coders scored the videos (100 and 18.42% of the videos, respectively). To ensure independence the second coder followed only written instructions. A time-unit kappa was calculated to estimate inter-observer agreement, defined as the accuracy of the overlap between the interval patterns generated by the raters for the same recording. The overlap between two strings of onset and offset points, generated by the two raters, was calculated as intra-class correlation (ICC). The overlap was high (ICC = 0.924). The analysis was conducted in R (v.3.5.1, the R Foundation for Statistical Computing: http://www.R-project.org). Significance level was set at 0.05 (two-tailed).

### Hypotheses

Regarding success in the test, we hypothesized that:

(H1A) The subjects would be more likely to solve the test task in the no-conflict and the conflict conditions, in which they received trainings on a different looking but relevant task (FOT), than in the control, in which they did not receive such a training. This would suggest that the subjects did not spontaneously solve the test in the no-conflict and the conflict conditions, but in fact needed the previous trainings to solve it.
(H1B) The subjects would be equally likely to ultimately solve the test task in the no-conflict and the conflict condition, like great apes in an analogical setup (Bobrowicz et al., 2020). This would show that Goffin's cockatoos not only can generalize a relevant cued experience (no-conflict) but can also do so despite an irrelevant and misleading cued experience (conflict). Generalizing the relevant experience would be indicative of memory retrieval (no-conflict), and overcoming the irrelevant, misleading experience would be indicative of exercising an executive function that resolves retrieval competition.

Regarding interactions executed by the subjects in the test, we hypothesized that:

(H2) The subjects that were trained would interact (a) more with the right tool, and (b) more with the relevant components of the test apparatus than non-trained control. Furthermore, even if the subjects that were trained failed the test, they would be less likely to engage in entirely unproductive interactions, interacting (c) less with the wrong tool and the irrelevant components of the test apparatus, than non-trained control.
(H3A) In the no-conflict condition, the successful subjects would be more likely to interact with the right tool and the relevant components of the test apparatus than the unsuccessful subjects. The successful subjects would also be less likely to interact with the right tool and the irrelevant components of the test apparatus than the unsuccessful subjects in the no-conflict condition. After only the training on the FOT that involved the right tool only, all subjects should focus on the right tool, but the unsuccessful ones may fail to inhibit interactions with the components that were relevant in the FOT, a different-looking task, but are now irrelevant in the test task.
(H3B) In the conflict condition, the successful subjects would be more likely to interact with the right tool and the relevant components than the unsuccessful subjects. After the training on the POT that involved using the right and the wrong tools on the relevant components, albeit paired with a now incorrect action, the unsuccessful subjects may fail to focus on applying the right tool to the relevant components of the test apparatus.
(H3C) The subjects that received only the training on the FOT would interact (a) more with the right tool than the subjects that received also the training on the POT, in which they used both the right and the wrong tools. Furthermore, the subjects that received only the training on the FOT would interact (b) less with the relevant components of the test apparatus than the subjects that received also the training on the POT, in which they interacted with the relevant components, albeit using another action. The successful subjects in the no-conflict condition would interact (c) more with the right tool and the irrelevant components of the test apparatus than in the conflict condition. Further, the successful subjects in the no-conflict condition would interact (d) less with the wrong tool and the relevant components of the test apparatus than in the conflict condition.
(H4A) In the no-conflict condition, successful subjects might start by applying the right tool to the irrelevant components (because these components were relevant in the FOT), and thereafter switch toward the relevant components. Other successful subjects may immediately identify the relevant components and apply the right tool to these components from the beginning to the end of the test. Testing this hypothesis may require comparing how interaction patterns in the test change over time for each individual. Individual interaction patterns in the Goffin's cockatoos would match individual interaction patterns in great apes on an analogical setup (Bobrowicz et al., 2020). This would suggest that similar cognitive functions could underlie performance on the task in Goffin cockatoos and great apes.
(H4B) In the conflict condition, the successful subjects might start by applying both the right and the wrong tool to the relevant components (as per POT), using an incorrect action associated with these tools in the POT, and thereafter switch to applying the right tool and the action trained in the FOT to the relevant components of the test apparatus. Testing this hypothesis may require comparing how interaction patterns in the test change over time for each individual. Individual interaction patterns in the Goffin's cockatoos would match individual interaction patterns in great apes on an analogical setup (Bobrowicz et al., 2020). This would suggest that similar cognitive functions could underlie performance on the task in Goffin's cockatoos and great apes.

### Statistical Analysis

Due to the low overall success rate in the test (control: *n* = 0/5, no-conflict: *n* = 3/7, conflict: *n* = 2/5), the power of relevant Generalized Linear Mixed Models would be too low, so the observed results were reported instead. We quantified the performance in the test in two ways. First, each bird received a score of 1 if it solved the test, and a score of 0 if it failed to do so. Second, the duration of interactions with the test task was calculated for each individual and compared subsequently across individuals and conditions. To trace whether there was a shift in engaging in various interactions over time, we quartered the overall interaction time in the analysis. In all figures showing this shift, individual datapoints and corresponding subjects' names are reported to highlight how such a shift unfolded across individuals (**Figures 5**, **6**).

To test hypotheses H1A-H1B, likelihood of success in each condition was calculated as a proportion of the number of successful birds to the number of all birds tested in a given condition. Likelihood of success on each task set was calculated as a proportion of the number of successful birds as compared to the number of all birds tested in the no-conflict and the conflict condition.

To test hypotheses H2-H4B, proportions of a given interaction time to overall interaction time were compared between conditions. Whenever these proportions were compared, medians (Mdn) and range (Minimum and Maximum values) of the proportions were reported, unless stated otherwise. Interpreting the results requires caution because in a small group of successful birds, three in the no-conflict and two in the conflict condition, and only one bird that completed both conditions, any outliers could substantially impact the mean proportion. Therefore, interaction patterns of the successful birds were reported individually.

For each training, we quantified how much time elapsed between the start of the first exposure to the FOT or the POT task and the end of the fifth successful attempt at retrieving a food reward from inside the respective puzzle box. These durations were compared across task sets to determine whether one of these sets was more difficult (e.g., motorically challenging) for the subjects than the other.

Because the useless tool was only briefly used by Fini and Kiwi (0.176 and 0.027 of the interaction time, respectively), interactions involving these tools were excluded from the analysis. Further, since Muppet and Muki refused to interact with the test task in the control condition, data from these subjects were removed from the dataset used in the interactions' analyses.

## Results

### Few Goffins Solved the Test in the No-Conflict and Conflict Conditions

Note that all conditions began with the baseline, in which subjects could spontaneously solve the test task. None of the subjects succeeded in the baseline on either of the sets, and none of the subjects succeeded in the control condition.

Note that in the control condition, subjects would attempt to solve the test task again after 24 h, without any trainings in between. In the no-conflict condition, subjects would receive a relevant training before attempting to solve the test, and in the conflict condition, subjects would receive a relevant training and an irrelevant training before such an attempt. Three out of seven subjects succeeded in the no-conflict condition, all on the hookset (Fini, Figaro, Pipin). Two out of five subjects succeeded in the conflict condition, one on the hookset (Figaro) and one on the screwset (Dolittle). Among birds that interacted with the test task in the test, interaction time varied between 9.38 and 185.56 s, and two birds in the control condition, Muppet and Muki, did not interact with the test task at all (all birds: Mdn = 30 s; without Muppet and Muki: Mdn = 37.35 s). Overall, the test, from presenting the subject with the test task to removing the test task, lasted between 50 and 1,362 s (Mdn = 408 s).

**Figure 3:**
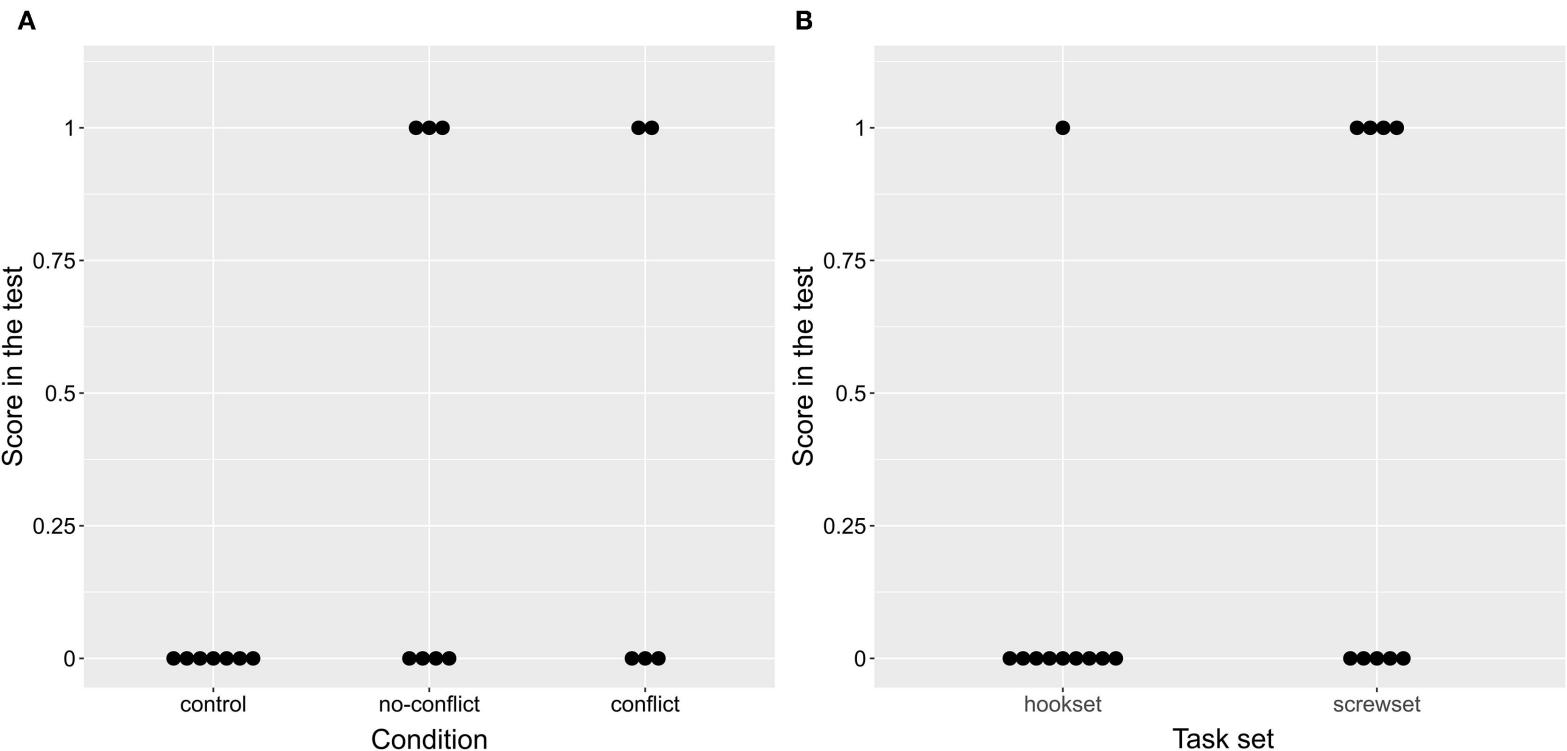
A plot of the effect of condition **(A)** and task set **(B)** on the score in the test. Note that **(B)** includes results of all three conditions.

Therefore, in line with hypotheses H1A-H1B, the birds were more likely to succeed in the two test conditions than in the control condition, although the likelihood was slightly higher for the no-conflict condition (no-conflict: 42.86%, conflict: 40%, control: 0%; Figure 3A). The birds were more likely to succeed on the screwset than on the hookset (screwset: 50%; hookset: 14.28%; Figure 3B). It is unlikely that this was caused by a difference in tool difficulty as there was no difference between training time spent on mastering the FOT task (Wilcoxon rank-sum test, *p* = 0.968) or mastering the POT task (Wilcoxon rank-sum test, *p* = 0.712) across the two task sets.

### Goffins Applied Tool Function to a Novel, Perceptually Dissimilar Task

In line with hypothesis H2, the training on the functionally overlapping task promoted interacting with the right tool in all subjects, regardless of the score in the test. The birds that trained on the FOT were more likely to use the right tool than those that did not (no-conflict: Mdn = 0.939, Min = 0.08, Max = 1; conflict: Mdn = 0.772, Min = 0.358, Max = 1; control: Mdn = 0, Min = 0, Max = 0.89). Furthermore, the birds that trained only on the FOT were more likely to use the right tool than the birds that were also trained on the POT, where they used both the right and the wrong tool (no-conflict: Mdn = 0.939, Min = 0.08, Max = 1; conflict: Mdn = 0.772, Min = 0.358, Max = 1).

**Figure 4:**
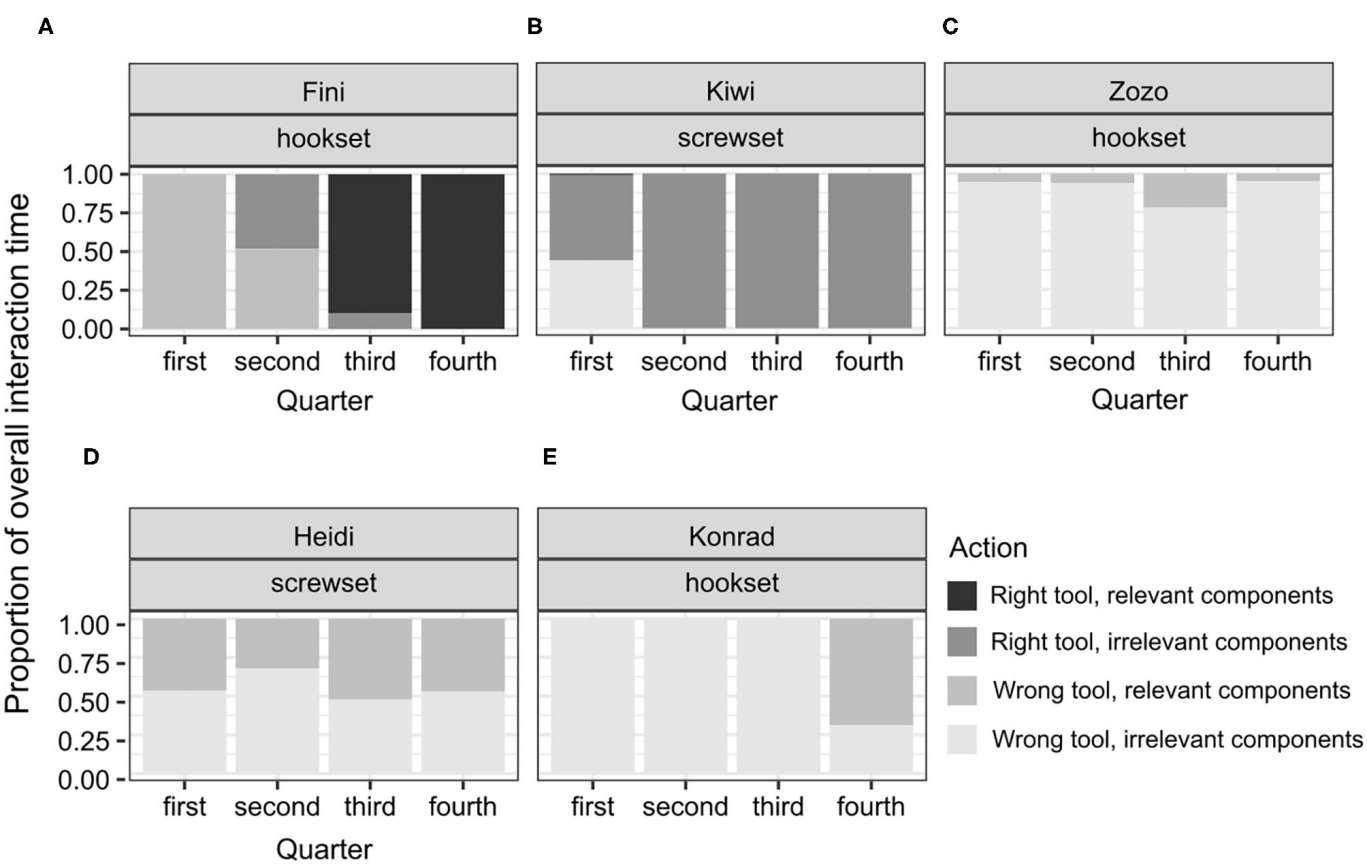
A plot of the proportions of individual interaction times to the overall interaction time throughout the test in the control condition **(A–E)**. Note that no subjects solved the test in this condition. This display is based on the observed proportions.

In line with hypothesis H2, the training on the functionally overlapping task promoted interacting with the relevant components of the test apparatus in all subjects, regardless of the score in the test. The birds that trained on the FOT were more likely to interact with the relevant components of the test apparatus than those that did not (no-conflict: Mdn = 0.406, Min = 0, Max = 0.742; conflict: Mdn = 0.525, Min = 0.049, Max = 0.975; control: Mdn = 0.172, Min = 0.002, Max = 0.854). Furthermore, the birds that received at least the training on the FOT were less likely to perform interactions between the wrong tool and the irrelevant components of the test apparatus (no-conflict: Mdn = 0.061, Min = 0, Max = 0.605; conflict: Mdn = 0.209, Min = 0, Max = 0.275; control: Mdn = 0.556, Min = 0, Max = 0.902; Figure 4A).

### The Unsuccessful Goffins Applied but Did Not Generalize Tool Function

In line with hypothesis H2, the unsuccessful birds in the no-conflict and the conflict conditions seemed to benefit from the FOT training, as they interacted less with the wrong tool and the irrelevant components of the test apparatus than the birds in the control condition (no-conflict: Mdn = 0.142, Min = 0, Max = 0,604; conflict: Mdn = 0, Min = 0, Max = 0.209; control: Mdn = 0.556, Min = 0, Max = 0,902; Figure 4B).

In line with hypothesis H3A, in the no-conflict condition, the successful birds interacted more with the right tool and the relevant components of the test apparatus than the unsuccessful birds (successful: Mdn = 0.406, Min = 0.066, Max = 0.584; unsuccessful: Mdn = 0.035, Min = 0, Max = 0.666). The successful birds also interacted less with the right tool and the irrelevant components of the test apparatus than the unsuccessful birds (successful: Mdn = 0.416, Min = 0.014, Max = 0.568; unsuccessful: Mdn = 0.533, Min = 0.221, Max = 0.939).

In line with hypothesis H3B, in the conflict condition, the successful birds interacted more with the right tool and the relevant components of the test apparatus than the unsuccessful birds (successful: Mdn = 0.33, Min = 0.207, Max = 0.452; unsuccessful: Mdn = 0.075, Min = 0.049, Max = 0.863). There was no difference in training time on the POT between the successful and the unsuccessful subjects (Wilcoxon rank-sum test, *p* = 0.685).

**Figure 5:**
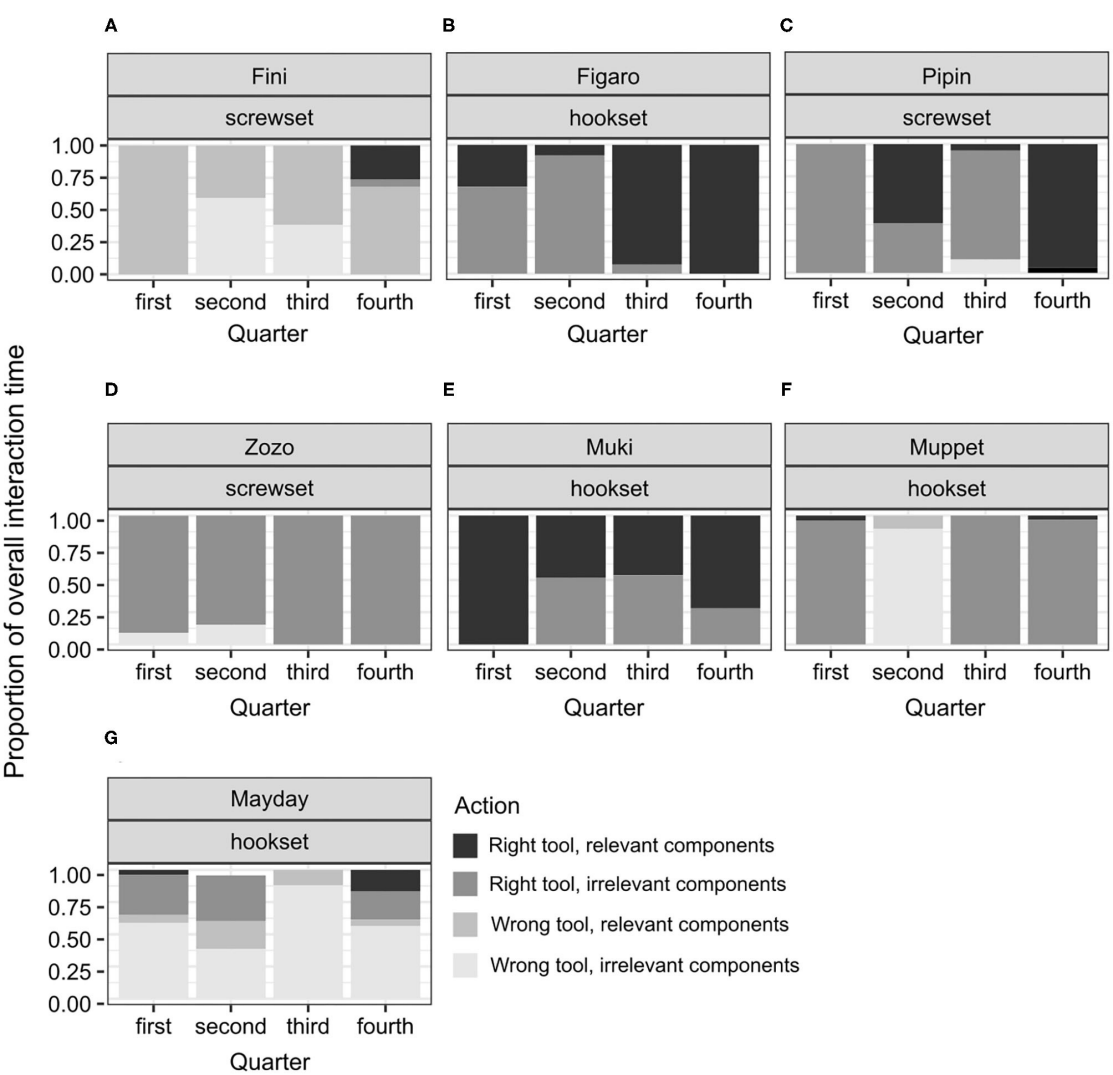
A plot of the proportions of individual interaction times to the overall interaction time throughout the test in the no-conflict condition in successful subjects **(A–C)**, and unsuccessful subjects **(D–G)**. This display is based on the observed proportions.

**Figure 6:**
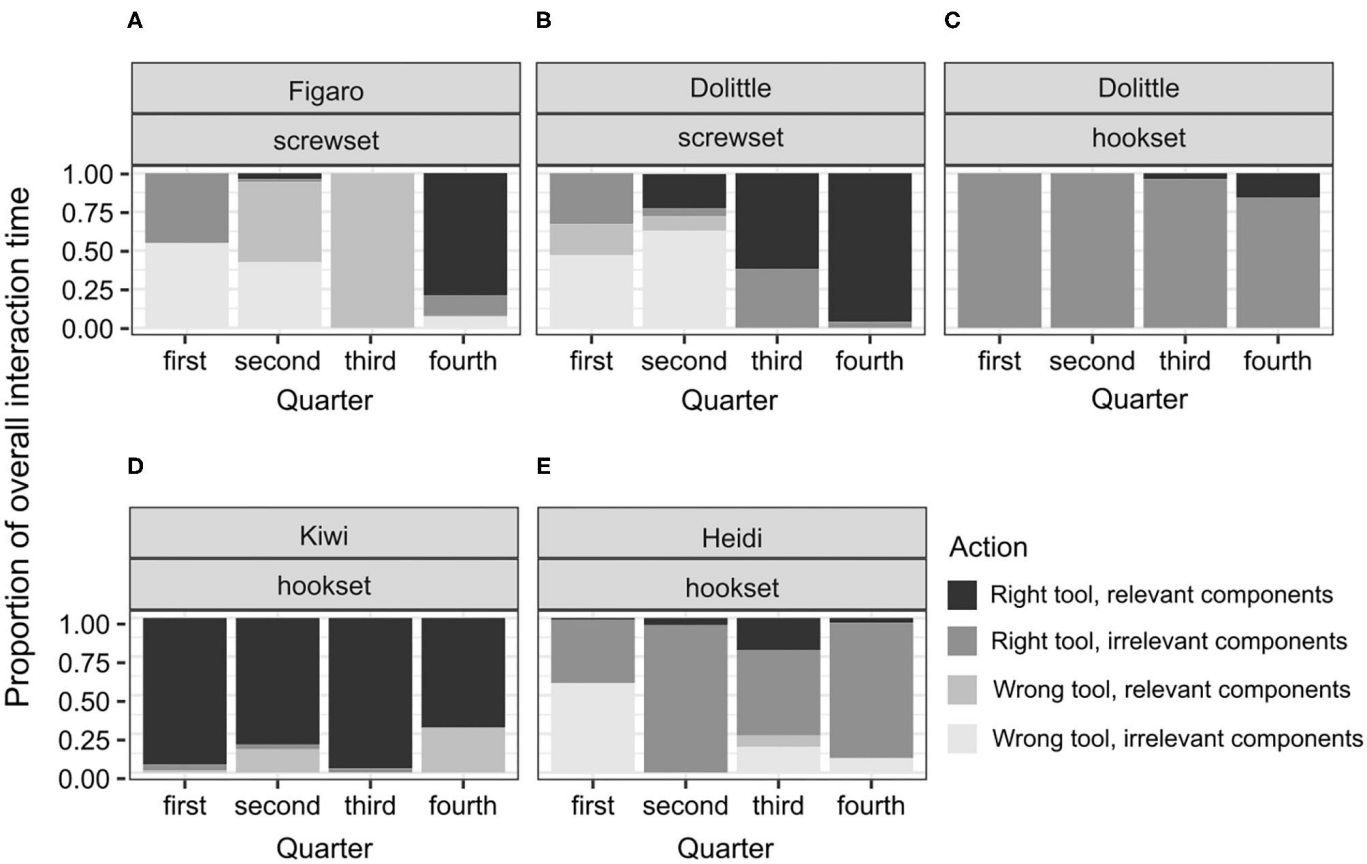
A plot of the proportions of individual interaction times to the overall interaction time throughout the test in the conflict condition in successful subjects **(A,B)**, and unsuccessful subjects **(C–E)**. This display is based on the observed proportions.

Among the unsuccessful birds, the variability of proportions was high across individuals and conditions (see Figures 4–**6**). This is not surprising, given that the duration of the test for the unsuccessful birds was not determined by individual's actions from picking the tool to solving the test. That is, interactions could not reflect the birds' planned behavior from identifying, through attempting, to reaching a common goal (solving the task) across the individuals. No clear shifts in interaction patterns could be observed. In each test condition, there was a group of birds that focused predominantly on the right tool, interacting both with the relevant and the irrelevant components of the test apparatus. This group comprised Zozo (Figure 5D), Muki (Figure 5E) and Muppet (Figure 5F) in the no-conflict condition and Dolittle (Figure 6C), Kiwi (Figure 6D) and Heidi (Figure 6E) in the conflict condition. All unsuccessful birds, albeit to a different extent, engaged in the following unproductive interactions: (1) incorrect action with the right tool and the relevant components, e.g., pushing the right tool sideways/downwards as in the POT, (2) correct action with the right tool but the irrelevant components, e.g., casting the tool on the wooden right-hand side of the test task (hookset) or bumping the tool against the lower part of the plexiglass front (screwset) as in the FOT, (3) combinations of incorrect actions, wrong tool and components of the test apparatus, both relevant and irrelevant. Some birds would unproductively execute the same actions with the same tool and the same components for several minutes in the test. For instance, in the no-conflict condition, Zozo and Muppet would keep using the right tool but performing the incorrect action on the irrelevant components of the test apparatus. In the conflict condition, Kiwi would keep executing the incorrect action with the right tool and the relevant component of the test task and Dolittle would keep attempting the correct action on the relevant components of the test apparatus, but with the wrong tool. The proportions of correct and incorrect actions are available in the Supplementary Information 4.

### Applying the Appropriate Tool Function Was More Straightforward in the No-Conflict Than in the Conflict Condition

Furthermore, in line with hypothesis H3C, the birds that trained only on the FOT were less likely to interact with the relevant components of the test apparatus than the birds that were also trained on the POT, where they interacted with the relevant components of the test apparatus, albeit using another action (no-conflict: Mdn = 0.406, Min = 0, Max = 0.742; conflict: Mdn = 0.525, Min = 0.049, Max = 0.975).

In line with hypothesis H3C, the successful birds interacted more with the right tool and the irrelevant components of the test apparatus in the no-conflict condition (Mdn = 0.416, Min = 0.014, Max = 0.568) than in the conflict condition (Mdn = 0.175, Min = 0.151, Max = 0.2) and less with the wrong tool and the relevant components of the test apparatus in the no-conflict condition (Mdn = 0, Min = 0, Max = 0.675) than in the conflict condition (Mdn = 0.227, Min = 0.073, Max = 0.381).

The successful birds were more likely to interact with the right tool and the relevant components of the test apparatus in the no-conflict than in the conflict condition (no-conflict: Mdn = 0.406, Min = 0.066, Max = 0.584; conflict: Mdn = 0.33, Min = 0.207, Max = 0.452). It must be noted that, first, among the successful birds, only Figaro completed both the no-conflict and the conflict conditions, and, second, that the observed proportions varied across individuals. Both Figaro and Pipin in the no-conflict condition interacted more with the right tool and the relevant components than Figaro and Dolittle in the conflict condition. This was consistent with the successful great apes' results (Bobrowicz et al., 2020). However, Fini, the other bird tested in the no-conflict condition, interacted far less with the right tool in the test. Overall, interaction patterns across the test differed between the no-conflict and the conflict condition in both the unsuccessful and the successful birds (Figures 4–6).

### The Successful Goffins Generalized Tool Function Despite Irrelevant, Distracting (No-Conflict) and Misleading (Conflict), Experiences

Because the successful bird groups comprised only three individuals in the no-conflict and two in the conflict condition, focusing on medians could overshadow the shift in interaction patterns. Therefore, individual proportions and comparing them to the proportions observed in the successful apes were used instead (Figures 5, 6, Supplementary Figure 4).

In the no-conflict condition, two out of three successful birds, Figaro and Pipin, in line with hypothesis H4A, would first engage mostly in interactions between the right tool and the irrelevant components of the test apparatus and would thereafter switch toward interactions between the right tool and the relevant components of the test apparatus. The other bird, Fini, would also switch toward such interactions but would initially apply both tools to the relevant components of the test apparatus. Namely, Fini kept interacting with the irrelevant components of the test apparatus, both with the right and the wrong tool, throughout the test. Only right before the solution, in the 4th quarter, did Fini interact with the right tool and the relevant components of the test apparatus. All Fini's interactions in the test lasted 10.69 s, that is, were half as long as Figaro's (24.06 s) and one quarter as long as Pipin's (40,535 s; see Supplementary Information 4). Figaro and Pipin focused on the right tool throughout the test, with Figaro focusing predominantly on the relevant components from the 3th quarter onwards and Pipin focusing predominantly on these components in the 4th quarter. Therefore, Figaro's pattern of interactions, that is, focusing initially on the right tool but the irrelevant components and thereafter on the right tool and the relevant components of the test apparatus, resembled that of Selma, a chimpanzee involved in the study with great apes (Supplementary Figure 4E). Pipin's pattern of interactions was different than in any of the apes: focusing initially on the right tool and the irrelevant components, then applying this tool to the relevant components, then the irrelevant again, and thereafter, to the relevant components soon before the solution. Taken together, the interaction patterns in the no-conflict condition point toward: (1) rapid, trial-and-error interactions in Fini's case, (2) cueing to irrelevant components, unproductive tool use and thereafter a switch toward the relevant components in Figaro's case, and (3) cueing to irrelevant components, then unproductive tool use, an unproductive switch toward the relevant components, a switch back to the irrelevant components, and only thereafter a switch toward the relevant components of the test apparatus in Pipin's case. Perhaps for this reason Pipin's overall interaction time was twice as long as Figaro's.

In the conflict condition, contrary to hypothesis H4B, Figaro and Dolittle, the two birds that solved the test did not immediately focus on the relevant components of the test apparatus and instead applied both the right and wrong tool to the irrelevant components. In line with the hypothesis, they did, however, switch to the right tool and the relevant components later in the test. Specifically, Figaro's and Dolittle's interaction patterns were similar in the 1st and the 4th quarter of the test, focusing predominantly on the interactions with both tools and the irrelevant components of the test apparatus in the 1st quarter and the interactions with the right tool and the relevant components in the 4th quarter (Figures 6A,B). Compared to the successful great apes, Figaro and Dolittle focused much more on the irrelevant components at the beginning of the test, both with the right and the wrong tool (Figures 6A,B, Supplementary Figure 4). In the 2nd quarter, Figaro and Dolittle interacted with the wrong tool and the irrelevant components far more than most of the apes (except for one female chimpanzee, Supplementary Figure 4F). In the 3rd quarter, Dolittle's interaction pattern resembled that of male great apes (Supplementary Figures 4H,I), that is, switching to interactions with the right tool, especially with the relevant components. Figaro's interaction pattern resembled that of a female chimpanzee, that is, focusing predominantly on the wrong tool and the relevant components. Soon before solving the test, both Dolittle and Figaro focused predominantly on the right tool and the relevant components, just like most of the great apes (except for a female chimpanzee, Supplementary Figure 4G). Taken together, the interaction patterns in the conflict condition point toward a similar start- and endpoint in Dolittle's and Figaro's test, but two different ways of getting from the start- to the endpoints. Namely, at a similar point along the way both Dolittle and Figaro identified the relevant components, but, while Dolittle used the right tool, Figaro used the wrong one. However, both of interaction patterns overlapped with those executed by great apes (Supplementary Figure 4).

## Discussion

Our findings suggest that proficient tool users among Goffins can use relevant past experiences that overlap with a novel task, even if the relevant experiences are followed by irrelevant ones. However, having relevant experiences may not suffice for solving the novel problem as only the most proficient tool-innovators successfully used them. Although all birds with relevant experience focused on the right tool, only some were able to generalize tool function, that is, apply the tool to the different-looking yet relevant aspects of the problem. In other words, the previous experiences were helpful only for those birds that exploited the overlap between these experiences and the novel problem, showing that solving the novel problem was not an inevitable result of the training(s). Note that the discussion of our findings regards the observed results and not statistically meaningful differences between the conditions.

The Goffins that solved the test despite having two conflicting experiences initially focused more on the irrelevant aspects of the problem than the Goffins that did not solve the test and the successful Goffins that had only the relevant experience. Therefore, exclusively the successful Goffins with two conflicting experiences seemed to be hindered by having the irrelevant, conflicting experience. Suffering an initial drop in performance is a measure of conflicting memories in humans (Sohn et al., 2003; Anderson, 2005) and is consistent with the behaviors of great apes in a similar task (Bobrowicz et al., 2020). However, in later stages, the successful Goffins with two conflicting experiences reached similar levels of attending to the relevant aspects of the problem as the successful Goffins that had only the relevant experience. That is, the successful individuals with two conflicting experiences likely suffered and resolved memory conflict. In this study, the irrelevant, misleading experience was always more recent than the relevant experience to ensure that the irrelevant traces would not have decayed before the test, and in fact be stronger. However, future studies could invert the order of experiences in the conflict condition to disentangle whether Goffins might adhere to information primacy, thereby overshadowing a recency effect, as has been shown to be the case in pigeons with longer retention latencies (Santiago and Wright, 1984; Wright et al., 1985). This should lead to a weaker or no memory conflict if the more recent experience is relied on, whereas the memory conflict would be increased in case greater focus is posed on the initial experience.

Although both Goffins and great apes resolved memory conflict and focused on the right tool and the relevant components right before solving the test, they demonstrated distinct interaction patterns along the way. Detecting the relevant components in the test was seemingly more difficult for the Goffins than for the apes, even those that only had a functionally relevant experience, suggesting that transfer of experience during tool innovations may be more challenging for the Goffins. Interestingly, however, having two conflicting experiences had a similar impact on the successful Goffins and the successful great apes, promoting focus on the irrelevant components of the test apparatus. This suggests that resolving memory conflicts may follow the same trajectory in both species. Although, in both species, resolving memory conflicts resulted in a shift from focusing on the irrelevant toward the relevant components of the test apparatus, tool use associated with these components may differ across individuals. Furthermore, due to a limited sample of the Goffins, caution is needed when interpreting the interaction patterns in the test, both in the no-conflict and the conflict condition. Especially in the no-conflict condition, these interaction patterns pointed to different steps in the birds' behavior, contingent upon different strategies, levels of understanding of tools' function and tool use experience.

In the conflict condition, the novel, test task cued retrieval of two previous, partly overlapping memories. The test task comprised the test apparatus and the three tools: the right, the wrong and the useless one. In other words, the test task comprised a certain spatial arrangement of features: apparatus components and tools. These features considerably overlapped with the features of the POT, which comprised the same spatial arrangement of features, both apparatus components and tools. Note that this entails the retrieval of the association (binding) between the right tool and the sliding action, and likewise, the wrong tool and the sliding action that was critical to solving the POT. If the subject was stuck on these associations and repeatedly executed the sliding action with this tool, it would not solve the test. Only if the subject could inhibit this association and search for other possibly applicable associations, would the subject retrieve the association between the right tool and the pulling action, formed when training on the FOT. Only then would this association be applied to now-relevant components of the test apparatus, perceptually different from the previously relevant components of the FOT. Training two different actions under different circumstances with the same, right tool is key to this study. Therefore, this study builds on the associative character of memory and accommodates associative learning accounts in interpretation of the results. We posit that the functional tool likely had a higher associative value in the test and therefore we do not use the preference for this tool as a measure of mnemonic conflict resolution. Instead, we focus on interactions between tools and features of the test apparatus because these interactions indicate which associations are currently applied by the bird to the task at hand. This was also the case in the study with great apes (Bobrowicz et al., 2020).

At first glance, the successful Goffins could have failed to encode or retain the irrelevant, distracting or misleading cues and therefore did not need to inhibit the irrelevant details in the test. It is possible that perceptual cues are not as strongly encoded as functional cues during problem solving. This could have been the case in the no-conflict, but not in the conflict condition. To solve the test in the no-conflict condition, the subjects must retrieve the relevant, functional aspects encoded in the FOT despite potentially distracting, consistently irrelevant perceptual details of the FOT. To solve the test in the conflict condition, the subjects must detect the relevant, functional aspects of the FOT despite potentially misleading, previously relevant but now irrelevant, perceptual details of the POT. In the conflict condition, at encoding, the tools and the relevant components of the apparatus are associated with a motor action that is relevant in the POT but will become irrelevant in the test. In other words, the perceptual features of the task are not only distracting as in the FOT; they are misleading because they were critical to the solution of the POT, but in the test task, they cue retrieval of an incorrect motor action. Hence, one must overcome such cueing and use a less recent motor action encoded in another task to solve the test.

The experimental setup used in this study relies on different degrees of perceptual similarity as this similarity is pitted against functional relevance. In principle, manipulating perceptual similarity can be problematic in animal cognition studies because it can fail to cue any relevant memories, if the perceptual overlap is too low, or spur a habitual, automatic response, if the perceptual overlap is so high that it cues only a single relevant memory (Povinelli and Henley, 2020). However, in the present study, this problem was avoided in the conflict condition through introducing high perceptual similarity between the POT and the test, and forcing the subject to, first, encode it on the POT and, second, disregard it in the test. The balance between sufficient perceptual similarity and sufficient perceptual dissimilarity is not, therefore, applicable to the conflict condition.

As mentioned in the introduction, the theoretical account of memory used in the study assumes that the relevance of information bits is guided by perceptual and functional similarity and is estimated across a pool of experiences available in subject's memory. Therefore, in principle, the subject's behavior in the test can be guided by the tasks involved in the study designed as well as by other tasks completed in the past. For this reason, we have included a baseline measurement in our design, in which the animal was exposed to the test task and could potentially solve it based on previous experiences. As none of the Goffins solved the test task at baseline and in the control condition, it seems that they did not have a sufficient physical knowledge to solve the test, unless they received the training on the FOT.

When exposed to the novel problem that cued the retrieval of two memories, a relevant and an irrelevant one, some individual cockatoos, like some great apes, could selectively retrieve these competing memories. In humans, and likely in great apes, resolving retrieval competition is mediated by the prefrontal cortex. In birds, this capacity is likely mediated by the nidopallium caudolaterale (NCL), an area analogous to the mammalian PFC. The NCL, like the PFC, mediates executive control of attention, memory and behavior (Güntürkün and Bugnyar, 2008; Herold et al., 2011; Herculano-Houzel, 2020). Such control may support high levels of flexibility that have independently evolved in distantly related species (Herold et al., 2011), and technical innovativeness, as all Goffins that resolved the conflict between the memories were proficient and innovative tool-users. However, as only 5 individuals solved the test, 2 of which succeeded in the conflict condition, it is difficult to generalize the results onto the general population of Goffin's cockatoos. This 2:5 ratio may differ in the general population of the species.

The unsuccessful birds were more persistent in their behavior, as they kept applying the right tool to now-irrelevant aspects of the problem because they fixated on the no-longer relevant aspects of the problem. This could be caused either by functional fixedness (Sayol et al., 2016; Harrison and Whiten, 2018) or difficulties in detecting the now-relevant aspects of the problem. This contrasted with the successful birds, which quickly suppressed such interactions in the test, suggesting a better ability to inhibit the repetition of unrewarded behaviors. However, Goffin's cockatoos have previously been tested on response inhibition tasks (Auersperg et al., 2014) and should be able to inhibit unproductive responses (Auersperg et al., 2008; Laumer et al., 2016). Both the response inhibition tasks and the present task required assessing tool functionality. However, contrary to the present task that required inhibiting an ongoing, unproductive response, the response inhibition tasks required inhibiting one of two viable responses ahead of issuing an alternative response. Inhibiting an already ongoing action and switching to a productive one may be more difficult than inhibiting a behavioral response ahead of acting. Overall, tool-using skills could have been helpful for solving the novel task, as the Goffins that succeeded in the test had a history of high-level performances across several innovative tool-use tasks (Pipin, Figaro, Dolittle: Auersperg et al., 2014; Fini: Laumer et al., 2017). However, other individuals that evinced tool manufacture and tool use did not succeed in our study (Kiwi: Auersperg et al., 2014; and Mayday: Laumer et al., 2017), suggesting that enhanced tool using skills are not the sole ingredient for success. To determine how tool-use proficiency contributed to the success in our task in the future, the tool-use problems could be substituted by problems requiring, e.g., haptic exploration.

Our results suggest that some Goffin's cockatoos could detect and exploit the functional overlaps between the novel problem and memories of other, similar problems. Such detection did not prevent the Goffins from referring first to the visually similar yet inapplicable memory, but later allowed for suppressing this memory in favor of another, more appropriate one. This shows that resolution of conflict between memories may belong to Goffins' executive function repertoire.

## Data Availability Statement

The original contributions presented in the study are included in the article/Supplementary Material, further inquiries can be directed to the corresponding author/s.

## Ethics Statement

Ethical review and approval was not required for the animal study because all subjects used in this experiment originate from accredited European breeders, have full CITES certificates and are officially registered (following the Austrian Animal Protection Act §25 - TschG. BGBl. 118) at the district's administrative animal welfare bureau (Bezirkshauptmannschaft St. Polten Schmiedgasse 4-6, A-3100; St. Polten, Austria). The housing conditions, described here and in detail in the Supplementary Information, are in accordance with the species-specific guidelines provided by the Austrian Federal Act on the Protection of Animals (Animal Protection Act -§24 Abs. 1 Z 1 and 2; §25 Abs. 3 – TSchG, BGBl. I Nr. 118/2004 Art. 2). Furthermore, as our experimental procedures were purely appetitive, strictly non-invasive and based exclusively on behavioral tests, they were classified as non-animal experiments in accordance with the Austrian Animal Experiments Act (§2. Federal Law Gazette No. 501/1989), according to which ethical approval is not required for this study. The birds are not wing-clipped and therefore partake in experiments voluntarily: they are called into the experimental chamber by name. Various fresh and cooked food sources and fresh water is always available *ad libitum* in our aviary. Specific treats (cashew nuts) are reserved for experiments.

## Author Contributions

KB conceived the study, analyzed the data, interpreted the data, and drafted the paper. KB, AA, and MO'H designed and implemented the experiment. KB and CC collected the data. KB, MO'H, AA, and MO revised the paper. All authors contributed to the article and approved the submitted version.

## Conflict of Interest

The authors declare that the research was conducted in the absence of any commercial or financial relationships that could be construed as a potential conflict of interest.
