# Supplementary material for: Goffin's Cockatoos (Cacatua goffiniana) Can Solve a Novel Problem After Conflicting Past Experiences
Source: Front Psychol. 2021 Jun 29;12:694719. doi: 10.3389/fpsyg.2021.694719 (PMC8275924; doi:10.3389/fpsyg.2021.694719)
Supplement: Supplementary file 1 [file Data_Sheet_1.docx]

Supplementary Information 1

# Supplementary Information on Goffin’s cockatoos

Goffin’s cockatoos are well-equipped for our study as they have efficient tool-handling techniques in the lab, face ecological demands on behavioral flexibility and are highly sensitive to novelty. First, they display elaborate beak-foot coordination which allows for precise tool manipulation with zygodactyl feet, upper mandible and strong thumb-like tongue (Auersperg et al. 2015, Luescher 2006; O’Hara et al. 2019). Second, they deal with different environments throughout the course of their lives, from agricultural habitats to monsoon forests, which likely put different demands on their cognitive capacities (Auersperg et al. 2015; Cahyadin et al. 1994; Jepson et al. 2001; Mioduszewska et al. 2018; O’Hara et al. 2019). And third, Goffins attend and flexibly respond to even minor changes in their environment (Auersperg, Kacelnik and von Bayern 2013; Auersperg, Laumer and Bugnyar 2013, 2016, 2017).

# Supplementary Method

## Participants

The subjects were aged between 7 and 11 years at the beginning of the study (Dolittle and Mayday: 7, Heidi, Kiwi, Konrad, Moneypenny, Muppet and Zozo: 8, Pipin: 10; Figaro and Fini: 11).

## Experimental setup

Dolittle was initially assigned to the conflict condition on the hookset, and the no-conflict condition on the screwset, but to compensate for some individuals’ dropout from the conflict condition on the screwset (Konrad, Mayday and Moneypenny), Dolittle was re-assigned to the under-represented conflict condition on the screwset. All data was included in the study and outliers were not detected.

## Apparatus


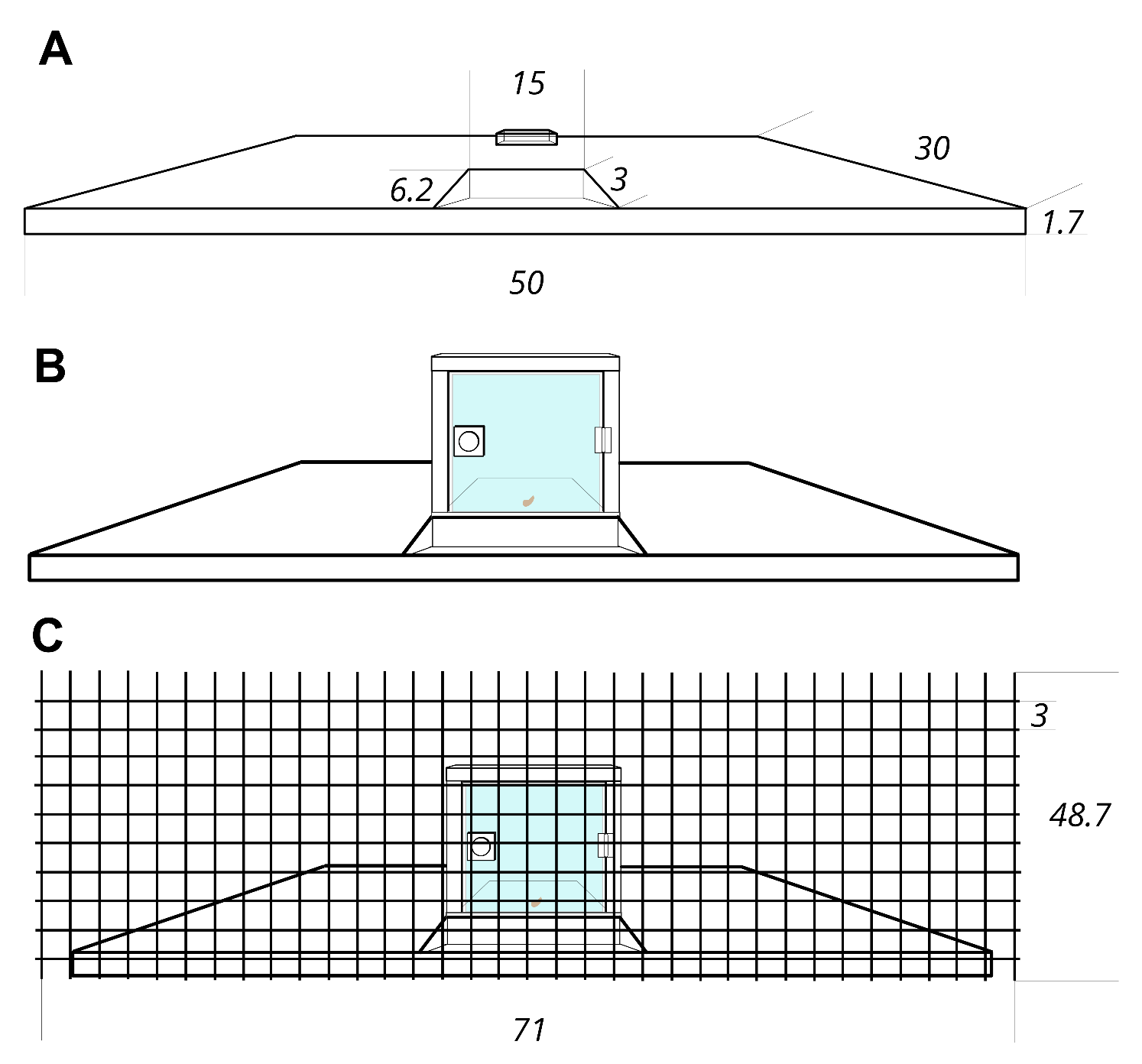


**Supplementary Figure 1.** A display of the experimental setup. (A-B) A wooden base was used to hold the apparatus in place. (C) The base and the apparatus were placed behind a mesh wall to prevent the subjects from haptic exploration of the apparatus. All dimensions were provided in [cm].

**Supplementary Table 1.** An overview of the subjects, sets, setups and tools used by each subject.

| **Name** | **Set** | **Setup** | | | **Tool material** | | |
| --- | --- | --- | --- | --- | --- | --- | --- |
|  |  | **baseline** | **training** | **test** | **baseline** | **training** | **test** |
| Dolittle | hookset | no cage | no cage, then cage | cage | wood+metal | wood+metal | wood+metal |
|  | screwset | cage | cage | cage | plastic | plastic | plastic |
| Fini | hookset | no cage | - | no cage | wood+metal | - | wood+metal |
|  | screwset | no cage | no cage | no cage | wood | wood | wood |
| Kiwi | hookset | no cage | no cage | no cage | wood+metal | wood+metal | wood+metal |
|  | screwset | cage | - | cage | wood | - | wood |
| Konrad | hookset | cage | cage | cage | wood+metal | wood+metal | wood+metal |
|  | screwset | cage | cage | - | clay | clay, then plastic | - |
| Mayday | hookset | no cage | cage | cage | wood+metal | wood+metal | wood+metal |
|  | screwset | cage | cage | - | wood | wood, then clay, then plastic | - |
| Muppet | hookset | no cage | no cage, then cage | cage | wood+metal | wood+metal | wood+metal |
|  | screwset | cage | - | cage | clay | - | clay |
| Figaro | hookset | cage | cage | cage | wood+metal | wood+metal | wood+metal |
|  | screwset | no cage | no cage, then cage | cage | wood | wood | wood |
| Heidi | hookset | cage | cage | cage | wood+metal | wood+metal | wood+metal |
|  | screwset | no cage | - | no cage | wood | - | wood |
| Moneypenny | hookset | cage | *-* | *-* | wood+metal | - | - |
|  | screwset | cage | cage | - | clay | clay, then plastic | - |
| Muki | hookset | cage | cage | cage | wood+metal | wood+metal | wood+metal |
|  | screwset | cage | - | cage | clay | - | clay |
| Pipin | hookset | cage | - | - | wood+metal | wood+metal | wood+metal |
|  | screwset | cage | cage | cage | clay | clay, then plastic | plastic |
| Zozo | hookset | cage | - | cage | wood+metal | - | wood+metal |
|  | screwset | cage | cage | cage | plastic | plastic | plastic |


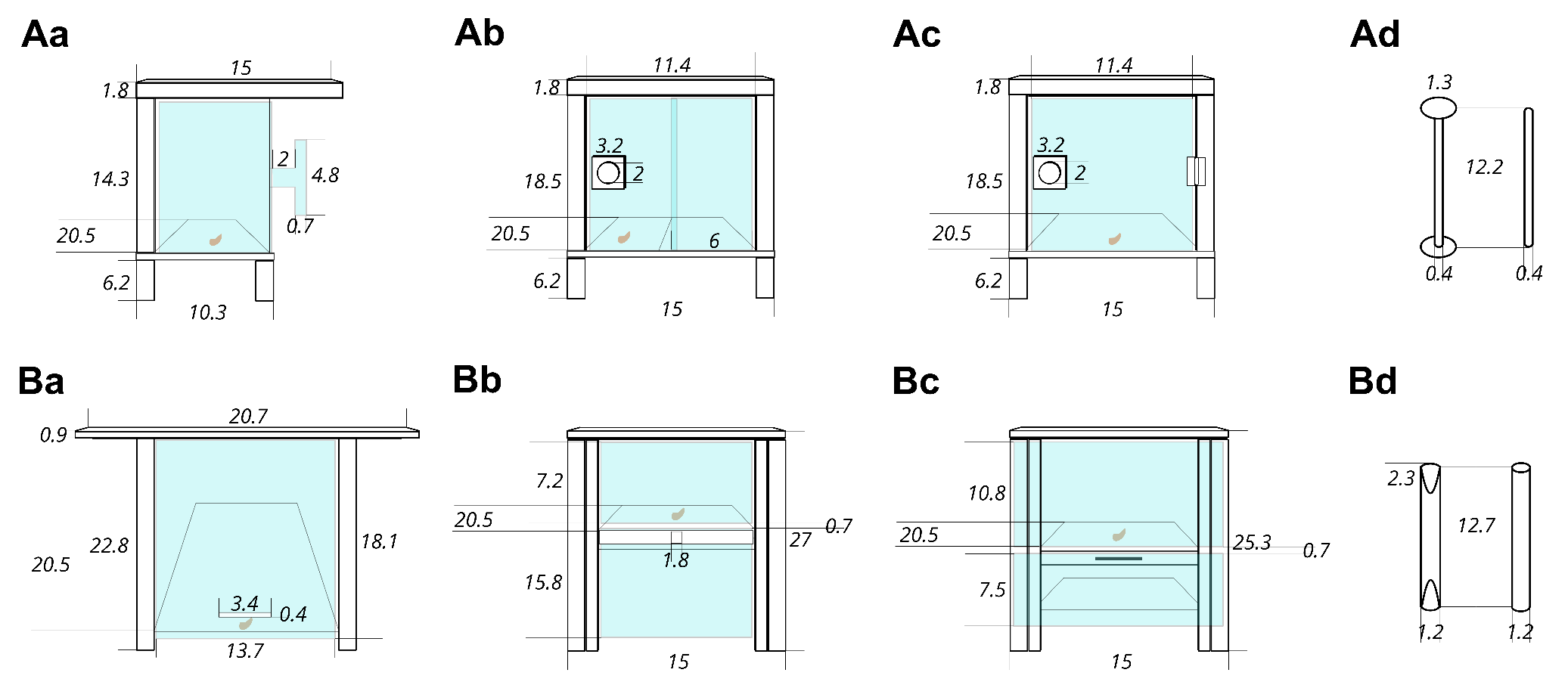


**Supplementary Figure 2.** A display of materials used in the study and their dimensions: (Aa) Hookset FOT, (Ab) Hookset POT, (Ac) Hookset Test, (Ad) Hookset tools, right and wrong, (Ba) Screwset FOT, (Bb) Screwset POT, (Bc) Screwset Test, (Bd) Screwset tools, right and wrong. All dimensions were provided in [cm].





**Supplementary Figure 3.** A display of relevant and irrelevant components of each apparatus. The relevant components are highlighted in green; all other components were considered irrelevant. (Aa-c) Hookset. (Ba-c) Screwset.

## Procedure

Although pre-defined time intervals for exposure in the baseline and in the test could have been specified, this approach would do less justice to the subjects’ performance than an exploratory approach. Therefore, in all conditions only the first five responses were recorded in the baseline, and in the test, regardless of the condition, the subjects could have interacted with all available tools and the apparatus unless they evinced the above-mentioned behaviours. By doing so, unwanted behaviours (e.g., apparatus destruction, leaving the experimental setup) were not reinforced, and frustration of the subjects as well as lack of cooperation with the experimenter in future encounters were minimized. ¨

**Supplementary Table 2. An overview of tool choices in the baseline.**

| **Name** | **Set** | **Tool** | **Choices out of 5** |
| --- | --- | --- | --- |
| Dolittle | hookset | right | 2 |
|  |  | wrong | 3 |
|  |  | useless | 0 |
|  | screwset | right | 3 |
|  |  | wrong | 2 |
|  |  | useless | 0 |
| Fini | hookset | right | 2 |
|  |  | wrong | 2 |
|  |  | useless | 1 |
|  | screwset | right | 2 |
|  |  | wrong | 3 |
|  |  | useless | 0 |
| Kiwi | hookset | right | 2 |
|  |  | wrong | 2 |
|  |  | useless | 1 |
|  | screwset | right | 3 |
|  |  | wrong | 2 |
|  |  | useless | 0 |
| Konrad | hookset | right | 2 |
|  |  | wrong | 3 |
|  |  | useless | 0 |
|  | screwset | right | 3 |
|  |  | wrong | 2 |
|  |  | useless | 0 |
| Mayday | hookset | right | 1 |
|  |  | wrong | 4 |
|  |  | useless | 0 |
|  | screwset | right | 1 |
|  |  | wrong | 4 |
|  |  | useless | 0 |
| Muppet | hookset | right | 4 |
|  |  | wrong | 1 |
|  |  | useless | 0 |
|  | screwset | right | 1 |
|  |  | wrong | 2 |
|  |  | useless | 3 |
| Figaro | hookset | right | 1 |
|  |  | wrong | 3 |
|  |  | useless | 1 |
|  | screwset | right | 2 |
|  |  | wrong | 1 |
|  |  | useless | 2 |
| Heidi | hookset | right | 1 |
|  |  | wrong | 3 |
|  |  | useless | 1 |
|  | screwset | right | 1 |
|  |  | wrong | 3 |
|  |  | useless | 1 |
| Moneypenny | hookset | right | - |
|  |  | wrong | - |
|  |  | useless | - |
|  | screwset | right | - |
|  |  | wrong | - |
|  |  | useless | - |
| Muki | hookset | right | 3 |
|  |  | wrong | 1 |
|  |  | useless | 1 |
|  | screwset | right | 0 |
|  |  | wrong | 0 |
|  |  | useless | 0 |
| Pipin | hookset | right | - |
|  |  | wrong | - |
|  |  | useless | - |
|  | screwset | right | 1 |
|  |  | wrong | 1 |
|  |  | useless | 3 |
| Zozo | hookset | right | 1 |
|  |  | wrong | 2 |
|  |  | useless | 2 |
|  | screwset | right | 2 |
|  |  | wrong | 3 |
|  |  | useless | 0 |

**Supplementary Table 3. Reasons for termination of the second exposure.**

| **Name** | **Set** | **Reason for termination** |
| --- | --- | --- |
| Dolittle | hookset | repeated unproductive motor movements on irrelevant components for a vast majority of the interaction time (96.5%) |
|  | screwset | solved the apparatus |
| Fini | hookset | removed all tools |
|  | screwset | solved the apparatus |
| Kiwi | hookset | engaged in apparatus destruction |
|  | screwset | repeated unproductive motor movements on irrelevant components for a vast majority of the interaction time (99.8%) |
| Konrad | hookset | engaged in tool destruction |
|  | screwset | - |
| Mayday | hookset | repeated unproductive motor movements on irrelevant components for a vast majority of the interaction time (83.1%) |
|  | screwset | - |
| Muppet | hookset | repeated unproductive motor movements on irrelevant components for a vast majority of the interaction time (94.3%) |
|  | screwset | refused to participate on three separate occasions |
| Figaro | hookset | solved the apparatus |
|  | screwset | solved the apparatus |
| Heidi | hookset | repeated unproductive motor movements on irrelevant components for a vast majority of the interaction time (91.6%) |
|  | screwset | removed all tools |
| Moneypenny | hookset | - |
|  | screwset | - |
| Muki | hookset | repeatedly attempted to leave the cage |
|  | screwset | repeatedly attempted to leave the cage |
| Pipin | hookset | - |
|  | screwset | solved the apparatus |
| Zozo | hookset | repeated unproductive motor movements on irrelevant components for a vast majority of the interaction time (91.5%) |
|  | screwset | repeated unproductive motor movements on irrelevant components for a vast majority of the interaction time (100%) |

## Familiarisation and habituation

At the beginning of the familiarization to the tools, the subject was called out by name from its social group and placed in a familiar perching spot. Then the tools from a respective set were presented by the experimenter one after another, and the subject was supposed to grab one end of the tool. If the subject began to freeze or move away from the tool as it got closer, the tool was put back, and either another, already familiar tool was presented in the same manner, or the familiarization was terminated for the day. This procedure was repeated until the subject would start reaching out with the beak to each of the tools. Only when the subject reached out to each tool five times in a row, did it proceed to the baseline. During the familiarization, the subjects were randomly rewarded with food items (pieces of a cashew nut), for instance, they received rewards even if they were too neophobic to reach out to the tool.

Afterwards, these subjects that participated in the task in the cage, proceeded to the habituation to the cage. At the beginning of the habituation, the subjects remained in a familiar perching spot while the tools were inserted into the cage. Then the subject was allowed to enter the cage and interact with the tools inside. Again, if the subject was reluctant to enter the cage, the experimenter encouraged the subject to enter with food items, tool play, and inserting own arms into the cage, on which the subject could walk freely and explore the cage. This was repeated until the subject was able to stay in the cage on its own without freezing or trying to escape the cage.

## Trainings

During the trainings on the FOT and the POT, the subjects learned how to execute a three-action motor pattern with given tools to release the food item from the given apparatus. As the subjects were trained in bartering in another experiment before (Laumer et al., 2020), they readily dropped the tool or passed the tool through the mesh to the experimenter, when she held her hand close to the mesh in a palm-up position. The experimenter did this whenever the subject did not succeed in opening the apparatus despite repeated interactions. After obtaining the tool, the experimenter would again demonstrate the correct solution. This sequence was repeated until the subject was able to execute a correct response without the experimenter’s help. If the subject was not able to execute a correct response upon the next attempt, the experimenter demonstrated the solution again, and this procedure was repeated until the subject was able to release the food item five times in a row. The experimenter made sure that the subject learned to use both the right and wrong tool in the POT, and that it used each of the tools in the five criterion trials.

## Coding

A time-unit kappa was computed to estimate inter-observer agreement, understood as the accuracy of the overlap between the interval patterns generated by the raters for the same recording. As time-unit kappa succeeds in “giving credit for near misses and increasing the magnitude of κ” (Bakeman et al. 2009), it may provide an accurate picture of the agreement whenever time intervals are compared between coders. Each of the recordings was divided into consecutive one-second intervals, and for each interval a 0-1 response was determined. Occurrence of coding on the coder’s part was counted as 1, and its lack was counted as 0. The 0-1 responses for each interval were subsequently assembled into a coder-specific pattern, and finally, an inter-rater kappa coefficient was calculated between these two patterns.

Coding was terminated either with the offset of the last recorded interaction or with the offset of the first interaction that led to food item’s release. For each recording, several variables were computed from the coded intervals. To obtain these variables, certain interaction times were divided either by the overall time between the onset of the first interaction and the offset of the last interaction or by a half of this overall time. For a full list of variables see Table S4.

**Supplementary Table 4.** Equations for the interaction variables. X stands for a fourth of the overall interaction time in the test, all attempts included. Each letter represents time spent on certain interactions in [s].

| **No.** | **Variable** | | | **Equation** | **Definition** |
| --- | --- | --- | --- | --- | --- |
|  | **tools** | **components** | **time** |  |  |
| **1** | right | relevant | overall | (a+e+i+m)/4X | a proportion of time spent on interactions between a right tool and relevant components of an apparatus to overall time spent on interactions between all tools and all components of the apparatus |
| **2** | right | irrelevant | overall | (b+f+j+n)/4X | a proportion of time spent on interactions between a right tool and irrelevant components of an apparatus to overall time spent on interactions between all tools and all components of the apparatus |
| **3** | wrong | relevant | overall | (c+g+k+o)/4X | a proportion of time spent on interactions between a wrong tool and relevant components of an apparatus to overall time spent on interactions between all tools and all components of the apparatus |
| **4** | wrong | irrelevant | overall | (d+h+l+p)/4X | a proportion of time spent on interactions between a wrong tool and irrelevant components of an apparatus to overall time spent on interactions between all tools and all components of the apparatus |
| **13** | right | relevant | 1st quarter | a/X | a proportion of time spent on interactions between a right tool and relevant components of an apparatus to overall time spent on interactions between all tools and all components of the apparatus in the 1st quarter of a test |
| **14** | right | irrelevant | 1st quarter | b/X | a proportion of time spent on interactions between a right tool and irrelevant components of an apparatus to overall time spent on interactions between all tools and all components of the apparatus in the 1st quarter of a test |
| **15** | wrong | relevant | 1st quarter | c/X | a proportion of time spent on interactions between a wrong tool and relevant components of an apparatus to overall time spent on interactions between all tools and all components of the apparatus in the 1st quarter of a test |
| **16** | wrong | irrelevant | 1st quarter | d/X | a proportion of time spent on interactions between a wrong tool and irrelevant components of an apparatus to overall time spent on interactions between all tools and all components of the apparatus in the 1st quarter of a test |
| **17** | right | relevant | 2nd quarter | e/X | a proportion of time spent on interactions between a right tool and relevant components of an apparatus to overall time spent on interactions between all tools and all components of the apparatus in the 2nd quarter of a test |
| **18** | right | irrelevant | 2nd quarter | f/X | a proportion of time spent on interactions between a right tool and irrelevant components of an apparatus to overall time spent on interactions between all tools and all components of the apparatus in the 2nd quarter of a test |
| **19** | wrong | relevant | 2nd quarter | g/X | a proportion of time spent on interactions between a wrong tool and relevant components of an apparatus to overall time spent on interactions between all tools and all components of the apparatus in the 2nd quarter of a test |
| **20** | wrong | irrelevant | 2nd quarter | h/X | a proportion of time spent on interactions between a wrong tool and irrelevant components of an apparatus to overall time spent on interactions between all tools and all components of the apparatus in the 2nd quarter of a test |
| **21** | right | relevant | 3rd quarter | i/X | a proportion of time spent on interactions between a right tool and relevant components of an apparatus to overall time spent on interactions between all tools and all components of the apparatus in the 3rd quarter of a test |
| **22** | right | irrelevant | 3rd quarter | j/X | a proportion of time spent on interactions between a right tool and irrelevant components of an apparatus to overall time spent on interactions between all tools and all components of the apparatus in the 3rd quarter of a test |
| **23** | wrong | relevant | 3rd quarter | k/X | a proportion of time spent on interactions between a wrong tool and relevant components of an apparatus to overall time spent on interactions between all tools and all components of the apparatus in the 3rd quarter of a test |
| **24** | wrong | irrelevant | 3rd quarter | l/X | a proportion of time spent on interactions between a wrong tool and irrelevant components of an apparatus to overall time spent on interactions between all tools and all components of the apparatus in the 3rd quarter of a test |
| **25** | right | relevant | 4th quarter | m/X | a proportion of time spent on interactions between a right tool and relevant components of an apparatus to overall time spent on interactions between all tools and all components of the apparatus in the 4th quarter of a test |
| **26** | right | irrelevant | 4th quarter | n/X | a proportion of time spent on interactions between a right tool and irrelevant components of an apparatus to overall time spent on interactions between all tools and all components of the apparatus in the 4th quarter of a test |
| **27** | wrong | relevant | 4th quarter | o/X | a proportion of time spent on interactions between a wrong tool and relevant components of an apparatus to overall time spent on interactions between all tools and all components of the apparatus in the 4th quarter of a test |
| **28** | wrong | irrelevant | 4th quarter | p/X | a proportion of time spent on interactions between a wrong tool and irrelevant components of an apparatus to overall time spent on interactions between all tools and all components of the apparatus in the 4th quarter of a test |

# Supplementary Results


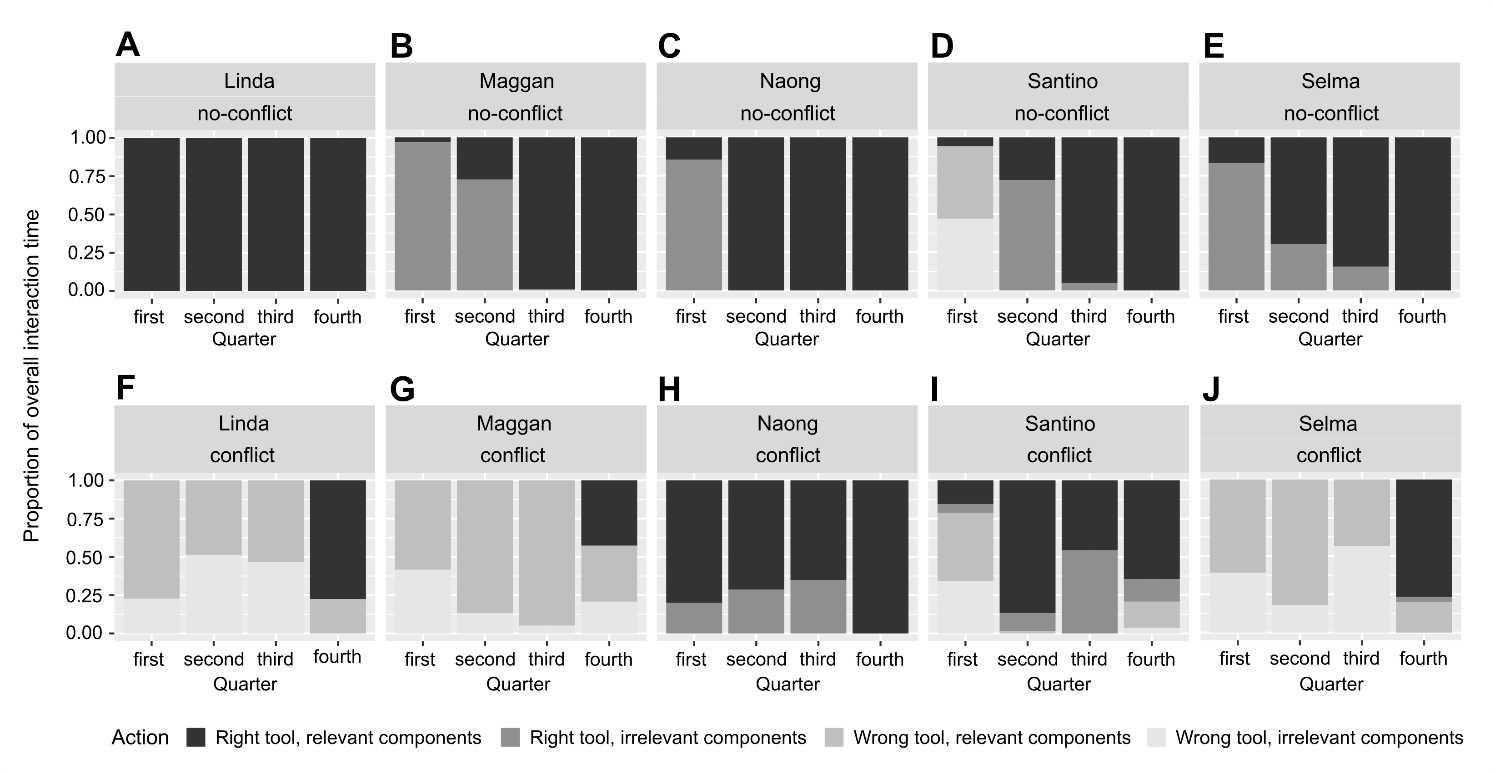


**Supplementary Figure 4.** A plot of the proportions of individual interaction times to the overall interaction time throughout the test in the no-conflict (A-E) and the conflict condition (F-J) in the successful great apes (Bobrowicz, Osvath and Johansson 2020). This display is based on the observed proportions.

## Supplementary References

Auersperg, A. M. I., Kacelnik, A. & Bayern A. M. P. v. 2013. ”Explorative learning and functional inferences on a five-step means-means-end problem in Goffin's cockatoos (Cacatua goffini)”. *PLoS ONE* 8: e68979.

Auersperg, A. M. I., Laumer, I. and T. Bugnyar. 2013. “Goffin cockatoos wait for qualitative and quantitative gains but prefer “better” to “more””. *Biol Letters* 9: 20121092.

Auersperg, A. M. I., Szabo, B., Bayern, A. M. P. v, and T. Bugnyar. (2014). “Object permanence in the Goffin cockatoo (Cacatua goffini*)*”*. J Comp Psychol* 128: 88–98.

Auersperg, A. M. I, Horik, J. O. v, Bugnyar, T., Kacelnik, A. Emery, N. J., and A. M. P. v. Bayern. 2015. “Combinatory actions during object play in psittaciformes (Diopsittaca nobilis, Pionites melanocephala, Cacatua goffini) and corvids (Corvus corax, C. monedula, C. moneduloides*)”. J Comp Psychol* 129: 62-71.

Auersperg, A. M. I., Borasinski, S., Laumer, I. & A. Kacelnik. 2016. Goffin's cockatoos make the same tool type from different materials. *Biol Letters* 12: 20160689.

Auersperg, A. M. I., Köck, C., Pledermann, A., O’Hara, M. & L. Huber. ”Safekeeping of tools in Goffin’s cockatoos (Cacatua goffiniana)”. *Animal Behav* 128: 125-33.

Bakeman., R., Quera, V., and A. Gnisci. 2019. “Observer-agreement for timed-event sequential data: A comparison of time-based and event-based algorithms”. *Behav Res Methods* 41: 137-47.

Bobrowicz, K., Johansson, M., and M. Osvath. 2020. “Great apes resolve competition between conflicting memories and selectively retrieve relevant memories to guide action”. *Sci Rep* 10: 12603.

Cahyadin, Y. 1994. “The status of Cacatua goffini and Eos reticulata on the Tanimbar islands; a preliminary analysis of field data”. *1st Eastern Inonesian-Australian vertebrate fauna*. Manado, Indonesia: Western Australian Museum.

Jepson, P., Brickle, N. and Y. Cayadin. 2001. “The conservation status of Tanimbar corella and blue-streaked lory on the Tanimbar Islands, Indonesia: results of a rapid contextual survey”. *Oryx* 35: 224-33.

Laumer, I. B., Massen, J. J. M., Lorck-Tympner, M., Wakonig, B., Carminito, C., and A. M. I. Auersperg. 2020. “Tentative evidence for inequity aversion to unequal work‐effort but not to unequal reward distribution in Goffin's cockatoos”. *Ethology* 126: 185-194.

Luescher, A. U. 2006. “Manual of Parrot Behavior”. Blackwell Publishing.

Mioduszewska, B., O’Hara, M., Haryoko, T., Auersperg, A., Huber, L., and D. M. Prawiradilaga. 2018. “Notes on ecology of wild Goffin’s cockatoo in the late dry season with emphasis on feeding ecology”. *Treubia* 45: 83-100.

O’Hara, M., Mioduszewska, B., Haryoko, T., Prawiradilaga, D. M., Huber, L. and A. M. I. Auersperg. 2019. “Extraction without tooling around – The first comprehensive description of the foraging- and socio-ecology of wild Goffin’s cockatoos (Cacatua goffiniana)”. *Behaviour* 156: 661-690.
